# The Hidden Shunt: An Asymptomatic Atrial Septal Defect Revealed by Echocardiography in a High-Performance Teenage Football Player

**DOI:** 10.7759/cureus.99115

**Published:** 2025-12-13

**Authors:** Ghita Bennis, Oualid Mohammed Taibi, Sara Hafid, Ghali Benouna, Fatimazahra Merzouk

**Affiliations:** 1 Cardiology, Cheikh Khalifa International University Hospital, Mohammed VI University of Health Sciences (UM6SS) Mohammed VI Foundation of Health Sciences, Casablanca, MAR; 2 Cardiology, Mohammed VI International University Hospital, Mohammed VI University of Health Sciences (UM6SS) Mohammed VI Foundation of Health Sciences, Casablanca, MAR

**Keywords:** adolescent athlete, atrial septal defect secundum, percutaneous atrial septal defects closure, sports cardiology, transthoracic echocardiograhy

## Abstract

Pre-participation cardiovascular evaluation aims to detect occult cardiac conditions that may increase the risk of sudden cardiac events in young athletes. Although echocardiography is not universally recommended for systematic screening, it can reveal silent congenital anomalies, particularly in highly trained individuals. In this report, we describe a case of a 14-year-old competitive football player with no prior medical history who underwent routine pre-participation evaluation. Clinical examination and resting electrocardiogram were normal. Transthoracic echocardiography (TTE), however, revealed an ostium secundum atrial septal defect (ASD) with mildly enlarged right chambers and a Qp/Qs ratio of 1.7, in the absence of pulmonary hypertension. Transesophageal echocardiography (TEE) confirmed adequate rims for device closure. Percutaneous occlusion using an ASD device was successfully performed without complications. Follow-up at one and three months confirmed appropriate device positioning and regression of right-heart dilation.

This case highlights the potential role of echocardiography in uncovering silent congenital heart disease (CHD) in young athletes. Although routine TTE screening is not recommended for the general athletic population, selected high-level athletes may benefit from imaging to detect structural anomalies not identifiable clinically. The literature supports early identification and percutaneous treatment of significant ASDs to prevent long-term right-heart remodeling and adverse outcomes.

## Introduction

Pre-participation cardiovascular evaluation aims to identify silent cardiac abnormalities that may predispose young athletes to adverse events. The 2020 European Society of Cardiology (ESC) guidelines define an athlete as an individual engaged in regular structured training with participation in official sports competitions. Competitive and elite athletes typically engage in more than 6 to 10 hours of intensive training per week, respectively [1,2]. These individuals are exposed to high physiological demands, which may unmask or worsen underlying cardiac abnormalities. Although the prevalence of cardiac conditions associated with sudden cardiac death in apparently healthy young athletes is low, approximately 0.3%, the consequences of missed diagnoses can be severe [3].

Atrial septal defects (ASDs), particularly the ostium secundum type, represent a frequent form of congenital heart disease (CHD) persisting into adolescence. Such defects may remain clinically silent for years, as centrally located shunts generate minimal turbulence and right-heart compensatory mechanisms often prevent the development of audible murmurs or overt symptoms, allowing the lesion to remain unnoticed despite high training loads. Even when asymptomatic, significant ASDs may progressively lead to right-heart dilation and exercise intolerance [4].

Despite its ability to detect structural abnormalities not evident clinically, echocardiography is not currently recommended for systematic screening of all athletes due to insufficient evidence supporting universal use [5]. Nevertheless, selected high-level athletes may benefit from targeted imaging, especially when silent congenital lesions may influence sports eligibility or long-term prognosis.

We report the case of a healthy, asymptomatic 14-year-old elite football player in whom a hemodynamically significant ostium secundum ASD was incidentally identified during pre-participation evaluation, ultimately leading to successful percutaneous closure.

## Case presentation

A 14-year-old male football player was evaluated as part of routine pre-participation cardiovascular screening prior to national-level competitive activity. He trained more than 10 hours per week, corresponding to an “elite athlete” profile per ESC classification. He had no medical history, took no medications, and denied palpitations, chest discomfort, presyncope, or reduced exercise tolerance. Physical examination was entirely normal. No murmurs, fixed splitting of S2, heave, or signs of right-sided overload were appreciated. Peripheral pulses were symmetric and normal. No signs of connective tissue disease were present.

Electrocardiogram

The resting 12-lead ECG was normal, with no conduction abnormalities, axis deviation, atrial enlargement, incomplete right bundle branch block, or repolarization changes.

Transthoracic echocardiography

Given the athlete’s high level and the institutional screening protocol, transthoracic echocardiography (TTE) was performed. Ultrasound imaging identified an ostium secundum atrial septal defect (ASD). The evaluation of the right cavities revealed a mild dilation of the right atrium and right ventricle (RA area = 18 cm²; RV basal diameter = 40 mm), with normal function and normal pulmonary arterial pressure. LV size and function were normal, and no additional structural anomalies were identified. During exercise testing, the patient remained asymptomatic, and agitated saline contrast was not performed at initial assessment.

Transesophageal echocardiography

**Figure 1:**
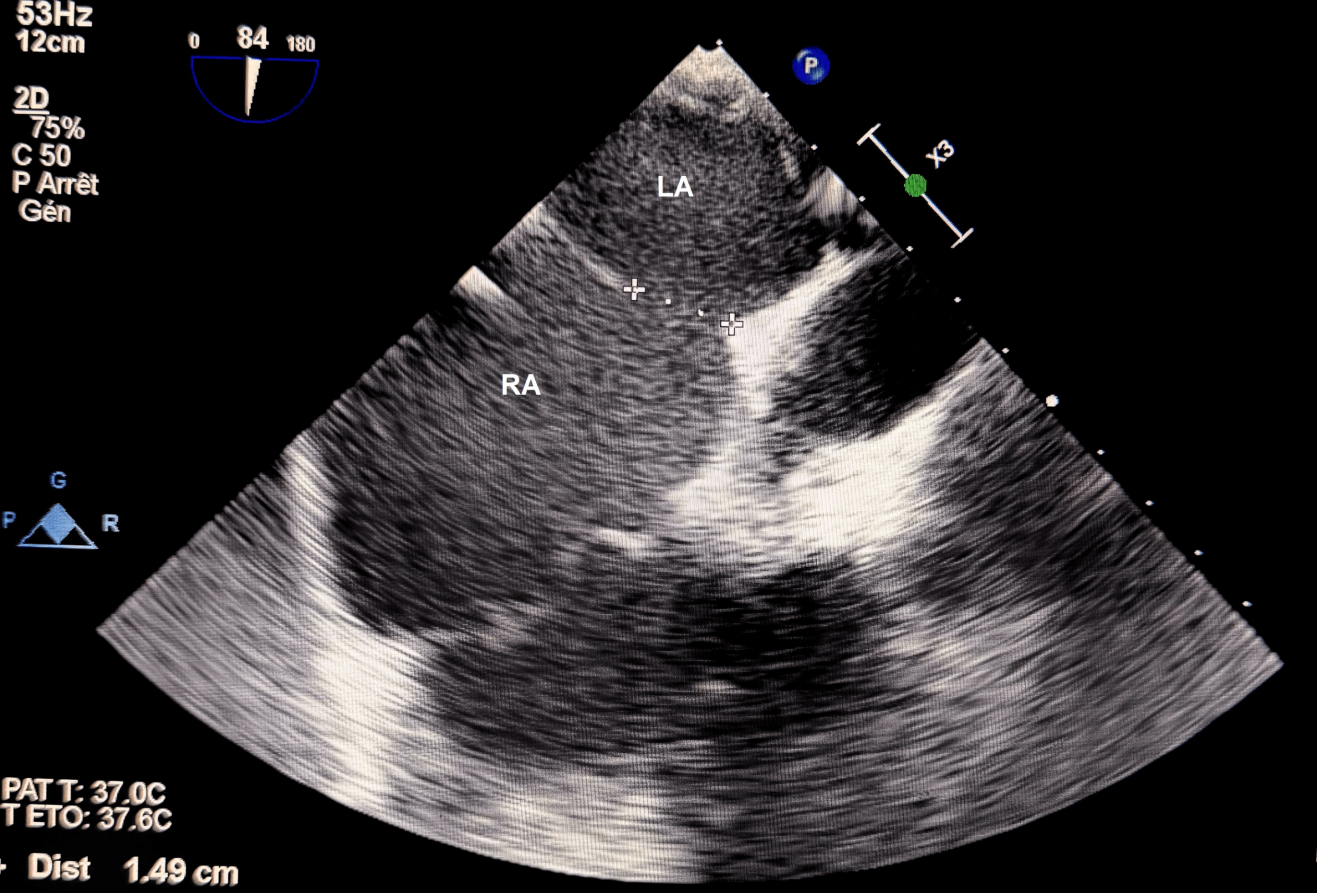
Transesophageal view showing the atrial septal defect (ASD) diameter RA: right atrium; LA: left atrium

To evaluate suitability for percutaneous closure, transesophageal echocardiography (TEE) was performed. It confirmed the centrally located ostium secundum ASD measuring 1.49 cm (Figure 1), with a hemodynamically significant left-to-right shunt, reflected by an estimated Qp/Qs ratio of 1.7 (Video 1), and well-defined and sufficient rims for device deployment. No associated anomalous pulmonary venous return or additional septal fenestrations were detected.

**Video 1:** Transesophageal color Doppler imaging showing the interatrial shunt

Intervention

Given the significant shunt (Qp/Qs ≥1.5) and evidence of right-heart dilation, percutaneous closure was recommended. Under general anesthesia and fluoroscopic-TEE guidance, an occluder device was successfully deployed across the defect (Video 2). Post-procedure imaging demonstrated immediate cessation of the left-to-right shunt with stable device position and no pericardial effusion.

**Video 2:** Fluoroscopic view of atrial septal defect device deployment

The patient was discharged on aspirin therapy for three months and advised a gradual return to training. Follow-up at one and three months confirmed appropriate device positioning and regression of right-heart dilation.

## Discussion

Transcatheter closure of secundum atrial septal defects has become the first-line therapeutic approach for most patients, combining high procedural success with low complication rates and favourable long-term outcomes [6,7]. In a multicentre cohort of more than 1300 children, percutaneous device closure achieved a success rate exceeding 95%, with only a minimal incidence of delayed major adverse events over a median follow-up of 3.5 years [6]. This favourable safety profile has also been reproduced in diverse clinical contexts, including resource-limited settings, where acute complications such as device embolization or atrioventricular valve interference remain similarly uncommon [8]. Furthermore, more recent data have extended these findings by demonstrating that percutaneous closure remains safe and effective across a broad spectrum of defect sizes, including moderate and even very large ASDs, with consistently low long-term complication rates [6].

Beyond procedural safety, one of the main advantages of ASD closure concerns the remodeling of the right heart. Left-to-right shunting through the defect imposes chronic volume overload on the right atrium and right ventricle, which may remain clinically silent for years but predispose one to long-term structural changes, arrhythmias, or right-heart failure if left untreated [9]. Multiple echocardiographic series demonstrate that right-sided volume overload begins to regress after device closure within the first month, with progressive normalization of right atrial and ventricular dimensions over three months [9,10]. Such reverse remodeling is often accompanied by improvements in right ventricular function, as assessed by modern imaging techniques such as speckle-tracking strain, and by normalization of electrocardiographic parameters, highlighting decreased ventricular strain after the elimination of the shunt [10,11]. Although right-heart remodeling generally progresses rapidly after closure, there are no explicit guideline recommendations defining the precise timing for resuming high-level competitive sports, and decisions remain individualized based on clinical reassessment.

In younger patients, including children and adolescents, these benefits are particularly relevant. Early closure of a hemodynamically significant ASD prevents the prolongation of right-heart overload through growth and maturation, possibly averting later complications such as atrial arrhythmias, exercise intolerance, or pulmonary vascular disease [7,12]. For athletic individuals, percutaneous closure provides not only anatomical correction, but also a physiological reset, improving right-heart hemodynamics while avoiding the morbidity associated with surgical repair.

In our case of a 14-year-old elite athlete, the ASD was asymptomatic, with normal physical examination and ECG, yet associated with a significant Qp/Qs and right-chamber dilation. The decision for percutaneous closure, therefore, appears justified, given the risk of silent remodeling and future adverse sequelae.

While resting ECG and clinical examination remain cornerstone tools [1], their sensitivity for congenital lesions is limited. Indeed, auscultation has a limited diagnostic yield, with the sensitivity of detecting a murmur in secundum ASDs reported to be below 50%, and resting ECG abnormalities present in only a minority of patients [13]. ASDs, especially centrally located secundum defects, often produce no murmur and may be completely silent until adolescence [14]. For this reason, echocardiography can reveal structural abnormalities that are otherwise undetectable with standard screening modalities. Despite these clear advantages, universal screening by echocardiography (TTE or TEE) in all athletes remains debated [5], as routine imaging in large athletic populations would pose logistical, economic, and ethical challenges. Instead, our experience and data from the literature support a selective screening strategy - targeting high-level athletes (e.g., elite, national-level, intensive training load) or those with clinical risk factors - to unmask silent congenital lesions that may impact long-term cardiac structure and performance.

## Conclusions

This case highlights the diagnostic value of transthoracic echocardiography in the pre-participation evaluation of young elite athletes. Although routine imaging in all athletes is not recommended, selective use of TTE in carefully chosen high-level competitors may help identify silent congenital lesions such as atrial septal defects and guide timely management. In our patient, identification of a significant ostium secundum ASD allowed timely percutaneous closure, preventing long-term complications and enabling safe continuation of competitive sport.

Early detection and minimally invasive closure represent an optimal strategy for managing asymptomatic secundum ASDs in adolescents, particularly those engaging in intensive athletic training. Future prospective studies focusing on outcomes in athletic populations are needed to refine guidelines and better define when selective screening may be appropriate.
